# 

**Published:** 1906-04

**Authors:** 


### BOOKS RECEIVED.

Case Teaching in Medicine. A series of graduated exercises in differential diagnosis, prognosis, and treatment of actual cases of disease. By Richard C. Cabot, A.B., M.D. (Harvard) Instructor in Medicine in the Harvard Medical School, and physician to out-patients at the Massachusetts General Hospital. Octavo, 220 pages. Boston: D. C. Heath & Co. 1906. (Price: Cloth, $1.50).

Transactions of the Section on Obstetrics and Diseases of Women, of the American Medical Association at the Fifty-sixth annual session, held at Portland, Ore., July 11-14, 1905. Chicago: American Medical Association Press. 1905.

Annual Report of the Surgeon General of the Public Health and Marine Hospital Service of the United States, for the fiscal year 1905. By Walter Wyman, Surgeon General. Washington: Government Printing Office. 1906.

Nursing; its Principles and Practice, for Hospital and Private use. By Isabel Hampton Robb, Graduate of the New York Training School for Nurses attached to Bellevue Hospital; late Superintendent of Nurses and Principal of the Training School for Nurses, Johns Hopkins Hospital, Baltimore, Md.; late Superintendent of Nurses, Illinois Training School for Nurses, Chicago, Ill.; Member of the Board of Lady Managers, The Lakeside Hospital, Cleveland, O.; Honorary Member of the Matron's Council, London, England. Third edition, revised and enlarged. Illustrated. 12mo. 565 pages. Cleveland: E. C. Koeckert. 1906.

A Textbook of Obstetrics. By Adam H. Wright, Professor of Obstetrics, University of Toronto; Obstetrician and Gynecologist to the General Hospital, Toronto, Canada. With two hundred and twenty-four illustrations in the text. Octavo, 591 pages. New York and London: D. Appleton and Company. 1905. (Price: cloth, $4.50).

The Examination of the Function of the Intestines by means of the Test-Diet. Its application in Medical Practice and its Diagnostic and Therapeutic value. By Prof. Dr. Adolf Schmidt, Physician-in-chief of the City Hospital Friedrichstadt in Dresden. Authorised Translation from the latest German Edition, by Charles D. Aaron, M.D., Professor of Diseases of the Stomach and Intestines in the Detroit Post-Graduate School of Medicine; Clinical Professor of Gastro-enterology in the Detroit College of Medicine; Consulting Gastro-enterologist to Harper Hospital, etc. With a frontispiece Plate in Colors. Crown Octavo, 91 pages, Extra cloth. F. A. Davis Company, Publishers, 1914-16 Cherry Street, Philadelphia. (Price: $1.00 net).

Progressive Medicine, Vol. VIII, No. 1, March 1906. A quarterly digest of Advances, Discoveries and Improvements in the Medical and

Surgical Sciences. Edited by Hobart Amory Hare, M.D., Professor of Therapeutics and Materia Medica in the Jefferson Medical College of Philadelphia. Octavo, 367 pages, 41 engravings, and 5 full-page colored plates. Per annum, in four cloth-bound volumes, $9.00; in paper binding, $6.00; carriage paid to any address. Lea Brothers & Co., Publishers, Philadelphia and New York.

Blakiston's Quiz Compends. A Compend of Obstetrics, especially adapted to the use of Medical Students and physicians. By Henry G. Landis, A.M., M.D., late professor of obstetrics and diseases of women in Starling Medical College, Columbus O. Revised and edited by William H. Wells, M.D., demonstrator of clinical obstetrics in Jefferson Medical College, Philadelphia, etc., etc. Eighth edition, illustrated. Philadelphia: P. Blakiston's Son and Co. 1906. (Price: $1.00 net).

